# Peripheral Artery Disease among a High-Risk Asian Population with Ischaemic Stroke, Cardiovascular Disease, or Diabetes Mellitus

**DOI:** 10.3390/jcm13133657

**Published:** 2024-06-22

**Authors:** Narayanaswamy Venketasubramanian, Koon Hou Mak, Keh Chuan Loh, John Tan

**Affiliations:** 1 Raffles Neuroscience Centre, Raffles Hospital, Singapore 188770, Singapore; 2 Mak Heart Clinic, Gleneagles Medical Centre, Singapore 258500, Singapore; makheartclinic@gmail.com; 3 Loh Keh Chuan Diabetes, Thyroid & Hormone Clinic, Mount Elizabeth Medical Centre, Singapore 228510, Singapore; info@endocrinology.com.sg; 4 The Vein Clinic & Surgery, Singapore 238859, Singapore; drjohntan@gmail.com

**Keywords:** peripheral artery disease, ischaemic stroke, cardiovascular disease, diabetes mellitus

## Abstract

**Background:** Peripheral artery disease (PAD) affects more than 100 million people globally. Most PAD studies have been performed among predominantly White populations—less is known about other ethnicities. The aim of this cross-sectional study was to determine the prevalence and risk factors of PAD in a high-risk Asian population with ischaemic stroke (IS), myocardial infarction, unstable angina (CVD), or diabetes mellitus (DM). **Methods:** Patients admitted for IS, CVD, or DM were recruited. Data were collected on age, sex, body mass index (BMI), index condition (CVD, IS, DM), history of hypertension, DM, hypercholesterolaemia, cigarette smoking, and claudication. The Edinburgh Claudication Questionnaire was administered, the ankle brachial index (ABI) was determined, and PAD was diagnosed if ABI was ≤0.9. **Results:** Of the 450 subjects recruited, 150 were placed in each index disease group, the mean age was 61.9 ± 10.32 years, 43.1% were female, and the mean BMI was 23.9 ± 4.3. Hypertension was reported in 59.3%, DM in 63.6%, hypercholesterolaemia in 39.6%, and smoking in 42.9% of patients. The prevalence of PAD was 27.1%, 22.0% in IS, 29.3% in CAD, and 30.0% in DM. PAD was associated with increasing age (adjusted odds ratio (aOR) 1.04/year, 95% confidence interval [CI] 1.01–1.06; *p* < 0.001), reduced BMI (aOR 0.94, 95% CI 0.89–0.99; *p* = 0.026), DM (aOR 1.59, 95% CI 1.20–3.18; *p* = 0.007), and hypercholesterolaemia (aOR 1.82, 95% CI 1.17–2.28; *p* = 0.007). It was more frequent in non-lacunar versus lacunar acute IS, non-ST segment elevation versus ST-segment elevation acute myocardial infarction, and insulin-treated versus non-insulin-treated DM. **Conclusions:** Our study showed a high prevalence of PAD among high-risk Asian patients. This was associated with increasing age, DM, and hypercholesterolaemia and inversely associated with BMI. Different rates were found in sub-groups of IS, CVD, and DM. Systematic approaches were used to identify these high-risk individuals and to improve their outcomes.

## 1. Introduction

Peripheral arterial disease (PAD) is atherosclerosis leading to the narrowing of the major arteries distal to the aortic arch [1]. In 2019, an estimated 113,443,016 individuals worldwide suffered from PAD, which is a 72% increase from 1990 [2]. The age-standardised prevalence per 100,000 in 2019 was 1402 per 100,000, while the age-standardised disability-adjusted life years lost (DALYs) attributable to PAD was 19.5 per 100,000, representing a 37% increase over 1990. The global burden due to PAD is expected to keep rising. A sex difference has also been noted, with prevalence and disability being higher in females, while mortality and years of life lost are greater among males. On a global basis, the number of people with PAD is highest in the Western Pacific region, intermediate in the Americas, Southeast Asia and Europe, and lowest in the Eastern Mediterranean region and Africa [3]. PAD prevalence is higher in the European Union than in other European countries [4] and in Black individuals compared to other ethnicities in the United States [5]. The burden of disease increases with the increasing sociodemographic index (SDI), with lower SDI regions undergoing a rapid growth of PAD-related burden compared to higher SDI regions [6]. These variations may be related to differences in the awareness, detection and control of vascular risk factors, accessibility to healthcare and differences in SD. But ethnicity may also have a role.

There are a number of well-recognised risk factors for PAD. These include advancing age (odds ratio OR 1.55 per 10-year increase, 95% confidence interval 95% CI 1.38–1.75), sex (OR 0.74, 95% CI 0.6–0.91 in males) diabetes mellitus (DM, OR 1.89, 95% CI 1.68–2.13), smoking (former smoking OR 1.70, 95% CI 1.39–2.09, current smoking OR 2.82, 95% CI 2.00–3.98), hypertension (OR 1.67, 95% CI 1.50–1.86), obesity (BMI > 30 kg/m^2^, OR 1.55, 95% CI 1.23–1.96) and hypercholesterolaemia (OR 1.34, 95% CI 1.17–1.53); it is also associated with stroke (OR 2.35, 95% CI 1.74–3.16) and coronary heart disease (OR 1.72, 95% CI 1.48–1.99) [3]. With the demographic trend of continued ageing in the world population and the projected rise in vascular risk factors, a greater burden of PAD can be expected in the foreseeable future.

The clinical spectrum of those with PAD ranges from being asymptomatic to claudication to critical limb ischaemia [7]. Effective therapies to reduce progression and/or limb loss include the cessation of smoking, exercise programs for claudication, lipid-lowering therapy, anti-thrombotic therapy with a single antiplatelet agent or combination aspirin with rivaroxaban, treatment of hypertension with an angiotensin-converting enzyme or angiotensin receptor blocker, grafts and endovascular techniques [8,9,10].

In addition to limb loss, PAD is associated with poorer prognosis, reduced quality of life and increased mortality [11]. It may also be a marker for atherosclerotic outcomes in other vascular beds [12]. Evidence of coexisting PAD is of prognostic value. In the international REACH registry, which enrolled patients from 44 countries (including Singapore) with established CVD, CeVD, PAD or with at least three atherothrombotic risk factors, the 3-year rate of myocardial infarction, stroke, vascular death or rehospitalisation for patients with symptomatic vascular disease in one vascular bed was 25.5% versus 40.5% among those with disease in multiple vascular beds, (*p* < 0.001) [13].

While claudication is a classical symptom of PAD, a careful history and physical examination are needed to rule out other causes of lower limb pain, including musculoskeletal and neurogenic causes [14]. The Edinburgh Claudication Questionnaire, an improved version of the World Health Organization (WHO)/Rose questionnaire, is a validated screening tool for PAD [15]. The ankle brachial index (ABI) is an inexpensive, non-invasive test where the ratio of the highest systolic blood pressure at the ankle to that of the brachial artery ≤ 0.9 is suggestive of PAD, with high sensitivity and specificity [16,17]. 

Most of the studies on PAD have been performed among white people—much less is known about the characteristics of this disease in other ethnicities [18]. Differing patterns of disease have been observed between Caucasians (more abdominal aortic aneurysms) and Black individuals and Asians (more distal arterial disease) [19]. Overall, the prevalence of PAD is higher in Black individuals [20] and lower in South Asians [21]. More data on Asian populations are needed.

There is an increased risk of PAD among those with stroke, CVD, or the aforementioned DM [3]. In a Malaysian study of 301 patients aged 32–90 years with established CVD, ischaemic stroke (IS) or DM, the overall prevalence of PAD was 23%, and it was 33%, 28% and 24% among patients with pre-existing CVD, IS and DM, respectively [22]. However, that study had small patient numbers and did not explore the risk ratios of PAD associated with the individual risk factors, nor among the subtypes of the disease groups investigated, to determine who was at particularly high risk of having PAD. 

The aim of this study was to determine the prevalence of PAD in a high-risk Asian population with IS, CVD or DM, the risk of its associated factors and investigate PAD within each of the three included disease groups.

## 2. Materials and Methods

The study was performed in Tan Tock Seng Hospital (TTSH), a public hospital located in central Singapore, serving the surrounding population of 1.4 million people.

Consecutive patients admitted to the Departments of Neurology for IS, Cardiology for myocardial infarction or unstable angina (CVD), or General Medicine with DM between 1 April and 30 September 2000 were approached to participate. Inclusion criteria included the following: diagnosis with IS, CVD or DM; informed consent obtainable from patients or their legally acceptable representative. Exclusion criteria were those who were aged less than 40 years, drowsy or aphasic throughout their hospital stay, or had amputations of both upper limbs or both lower limbs. Recruitment among the 3 disease groups was concurrent.

After obtaining informed consent from eligible subjects in the language they were conversant in, the trained study nurse administered a standardised questionnaire. Data were collected on subject demographics, age, sex, and index condition (IS, CVD, DM). Hypertension was diagnosed if there was a history of hypertension or if the patient had been prescribed medications for hypertension. DM was diagnosed if there was a history of DM or if the patient had been prescribed medications for DM. Hypercholesterolaemia was diagnosed if there was a history of hypercholesterolaemia or if the patient had been prescribed medications for hypercholesterolaemia. Cigarette smoking was diagnosed if the patient was a current or former smoker. Claudication was diagnosed if there was a history of calf pain on either side when walking, which was relieved by rest). The body mass index (BMI = weight/height^2^) was calculated. Subjects were then asked to fill in the Edinburgh Claudication Questionnaire. Finally, the ABI was determined in the right and left leg with the subject lying at rest—the highest systolic blood pressure (SBP) in the dorsalis pedis or posterior tibial artery of that lower limb was determined using the Doppler probe and was divided by the highest SBP after measuring in both brachial arteries using the standard auscultatory technique. PAD was diagnosed if ABI was ≤0.9 in either limb. There are recommendations to use ABI > 1.4 [23,24] or the toe-brachial index, especially among those with diabetes mellitus [17,25], to diagnose PAD. However, these parameters and indices were not used in the analysis of this study so as to allow comparisons of our findings with other studies discussed in this paper, as they largely used ABI < 0.9 to diagnose PAD. The research staff performing the ABI measurement were blinded to the other study information. 

Data were analysed using the Statistical Package for Social Sciences (SPSS) v25 (New York, NY, USA). Mean and standard deviations were calculated for normally distributed continuous variables, median and interquartile ranges for non-normally distributed continuous variables, and proportions for categorical variables. Significant differences in baseline characteristics were evaluated among those with and without PAD by univariable analysis, using the unpaired t-test for continuous variables and chi-square for categorical variables. Finally, multivariable analysis using logistic regression was performed using variables that were significantly associated with the univariable analysis for PAD. Statistical significance was taken at the *p* = 0.05 level.

Using the estimate of a prevalence of PAD of 25% found in the Malaysian study [22] and the range of 24–26% for a 95% CI, the sample size would be 451 subjects. Thus, the target was to recruit 450 subjects, 150 in each of the 3 groups of IS, CVD and DM. 

## 3. Results

The mean age of study subjects was 62 years, with fewer females than males and the described risk vascular factors (Table 1). The overall prevalence of PAD was 27.1%, higher than the symptoms of claudication or by the Edinburgh Questionnaire. By index disease, females outnumbered males only among those with DM, and hypercholesterolaemia and smoking were most frequent among those with CVD. Previous myocardial infarction was the least frequent among IS, while a previous stroke was the least common among those with CVD. While not statistically significant, PAD, as assessed by the symptoms of claudication, the Edinburgh Questionnaire and ABI, is highest among those with DM. Age and the number of years with DM were similar across all three index diseases. 

Within the index diseases, the frequency of PAD varied among the subtypes (Table 2). Among IS, PAD by ABI was found in 16.5% of lacunar infarction and 32.1% of non-lacunar infarction (*p* = 0.03). For CVD, PAD by ABI was found in 25.4% of unstable angina, 28.2% of ST segment elevation acute myocardial infarction, and 34.6% of non-ST-segment elevation myocardial infarction (*p* = 0.56). Among DM patients, PAD by ABI was found in 28.9% of non-insulin-treated diabetics and 60.0% of insulin-treated diabetics (*p* = 0.14). 

PAD occurred more frequently among older ages, females, and those with DM or hypercholesterolaemia; it was also associated with lower BMI (Table 3). However, the effect of sex was lost in multivariable analysis. The odds increased by 4% per year, and increasing age was nearly doubled by the presence of DM or hypercholesterolaemia and lowered by 6% for each increased unit of BMI. There was no significant effect on smoking status or index disease.

## 4. Discussion

Our study found that PAD was present in 27% of a high-risk Asian population with ischaemic stroke, cardiovascular disease, and diabetes mellitus. PAD was detected more frequently by ABI than symptoms of claudication or the Edinburgh questionnaire. Upon multivariable analysis, PAD was most strongly associated with increasing age, diabetes mellitus and hypercholesterolaemia and reduced with higher BMI.

The prevalence of PAD of 27.1% was comparable to the 25% found in the Malaysian study among similarly high-risk patients [22]. They found PAD in 23–28% of patients with IS, 33% of patients with pre-existent CVD, and 24% with DM; the corresponding frequencies in our study were 22.0%, 29.3%, and 30.0%, respectively. This is understandable as Malaysia and Singapore are neighbouring countries with Chinese, Malays and Indians as the main ethnic groups. 

PAD was detected in 22% of our IS patients. Among 1293 Korean patients with acute IS or a transient ischaemic attack (TIA), an ABI of ≤0.9 was found in 13.0% [26]. In a Pakistani study of 327 IS patients, the mean age was 57.6 ± 12.8 years, and PAD was identified in 18.3% of patients with ABI [27]. In Thailand, an ABI ≤ 0.9 was observed in 18.1% of 747 IS or TIA patients [28].

We identified PAD in 29.3% of our patients with CVD. Among 711 Korean patients, the mean age was 63.4 ± 11.0 years after undergoing percutaneous coronary intervention for CVD, and the prevalence of PAD was considerably lower, at 12.8% [29]. Approximately 38.4% of 117 Thai patients, with a mean age of 65 years, undergoing coronary angiography had PAD [30], while 19.1% of patients with coronary artery disease had PAD in another Singapore study [31].

PAD was diagnosed in 33% of our DM patients. In a multi-centre study of 6625 patients with DMs in Korea, China, Taiwan, Hong Kong, Indonesia, Thailand and the Philippines, the mean age was 63.7 ± 8.2 years, and the frequency of PAD was 17.7% using ABI ≤ 0.9 [32]. In a study of 3906 diabetics in Japan, the mean age was 60.8 years, and the frequency of PAD was 7.6% using a device “form PWV/ABI” [33]. In a Singapore study, 521 diabetics had a prevalence of PAD, defined as a resting ABI of <0.9 on either leg and/or a history of gangrene or non-traumatic amputation in 15.2% (95% CI, 12.3–18.5) [34]. As these studies are based on different disease populations, differing ages, and different diagnostic techniques to detect PAD, the prevalence of PAD among these studies is not directly comparable. Still, the prevalence of PAD in our study appears higher than in most Asian studies and is closest to that found in Malaysia. 

Our study found that among ISs, PAD via the ABI was found in 32.1% of patients experiencing non-lacunar infarction and 16.5% with lacunar infarction. In a study in China, 31.51% of patients with stroke from large artery atherosclerosis had PAD (ABI < 0.9), compared with 19.75% with small artery disease (*p* = 0.045) [35]. In a Korean study on IS, the prevalence of abnormal ABI was 18.4% in large artery atherosclerosis and 7% in small artery disease (*p* < 0.001) [26]. In the previously mentioned Thai study, abnormal ABI was more frequent among those with large artery disease (20.4%, *p* < 0.001) [28]

In our study, for CVD, PAD by ABI was found in 25.4% of unstable angina, 28.2% of ST-segment elevation acute myocardial infarction, and 34.6% of non-ST-segment- elevation myocardial infarction patients. In a French study, previous non-Q-wave myocardial infarction was associated with PAD (OR 1.50; 95% CI, 1.08 to 2.08; *p* = 0.02) [36].

Our study found strong associations between PAD and increasing age, DM and hypercholesterolaemia. This is consistent with the meta-analysis showing risk factors for PAD, including advancing age (OR per 10-year increase 1.55, 95% CI 1.38–1.75), DM (OR 1.89, 95% CI 1.68–2.13), and hypercholesterolaemia (OR 1.34, 95% CI 1.17–1.53); it also showed strong associations with smoking and hypertension [3]. While this study showed a non-significant trend among smokers, no association was found with hypertension—the reasons for these observations are unclear and would require further investigation. 

Of interest was our finding of an inverse association between PAD and BMI, consistent with the so-called ‘obesity paradox’, where PAD is less frequent among those with obesity. A recent review of studies on obesity and PAD had mixed findings [37]. Some studies found positive associations of PAD with a high BMI or upper body obesity or only among women; others showed that a high BMI or being overweight was a protective factor against PAD. In a study in China on 11,477 community-dwelling adults aged 40 years and above, for each standard deviation increase in the weighted BMI genetic risk score, the odds ratio (OR) for PAD was 1.17 (95% CI 1.07–1.27; *p* = 0.0004) [38]. However, a Thai study found that being overweight (body mass index [BMI] > 25 kg/m^2^, had a reduced risk of PAD (OR = 0.54, *p* < 0.05) [39]. 

Different studies have used differing criteria for the diagnosis of PAD. Clinically, while a single question on calf pain when walking can be asked, it is preferable that a validated questionnaire, such as the WHO/Rose or Edinburgh Claudication Questionnaire, be used. As an improved version of the World Health Organization (WHO)/Rose questionnaire, it is a validated screening tool for PAD with a sensitivity of 91.3% (95% CI, 88.1–94.5%) and specificity of 99.3% (95% CI, 98.9–100%) [15]. Where feasible, an objective assessment by the ABI would allow accurate documentation and the assessment of severity. The ABI is an inexpensive, non-invasive test, where the ratio of the highest systolic blood pressure at the ankle to that of the brachial artery ≤0.9 is suggestive of PAD, with a sensitivity of 61% (95% CI 55–69%), and specificity of 92% (95% CI, 89–95%) [16,17]. We note that among those diagnosed as having PAD on ABI, only 30.3% were positive on the question of intermittent claudication, and 19.7% by the questionnaire; among those without PAD, the proportions were 14.0% and 7.9%, respectively. In the Malaysian study, only 27% of those diagnosed as having PAD by ABI had symptoms [22]. This may be reflective of the subjective nature of questions and questionnaires, which are affected by interpretation by the patient—this further supports the use of an objective assessment using the ABI measurement to diagnose PAD.

In our study, the prevalence of PAD was highest using the ABI in all three disease groups. In view of the high prevalence, the consequences if left untreated and the availability of evidence-based interventions, detecting PAD by ABI should be part of the routine assessment of patients at risk of PAD.

The detection of PAD is important, not only for its presence. It may also be a marker for atherosclerotic outcomes in other vascular beds. Among those with established cerebro-(CeVD) or cardiovascular (CVD) disease, having PAD on top of this increases the risk of major cardiac events; in the EUCLID trial, compared to those with isolated PAD, the adjusted hazard ratios (aHR) were 1.34 (95% CI, 1.15–1.57) for PAD + CeVD, 1.65 (95% CI, 1.43–1.91) for PAD + CVD, and 1.99 (95% CI, 1.69–2.34) for PAD + CeVD + CVD [12].

With the rising numbers and ageing of world populations, the burden of PAD is likely to keep increasing. This can be counter-balanced by increasing efforts to effectively detect and optimally treat vascular risk factors—this will also help reduce the burden of other vascular diseases, such as stroke and CAD. Screening for PAD by asking the patient to fill in the Edinburgh questionnaire while sitting in the clinic waiting room, or even better, by trained healthcare professionals performing ABI at regular intervals among those at risk of PAD, should become part of the routine evaluation of our patients in primary care. A system should be in place to allow seamless referral for those suspected to have PAD to accessible specialists and teams interested and trained in the management of PAD. Public awareness of PAD can be raised by health education campaigns. 

Our study has some limitations. Although it was a single-centre study, and several other studies corroborated our findings, the sample size was modest—stronger associations may have been found with a larger study population. The study was performed on hospitalised patients, and the findings may not be generalisable to the community. ABI < 0.9 was used to diagnose PAD; however, an ABI > 1.4, or the toe-brachial index, especially among those with diabetes mellitus, have been proposed. Thus, the prevalence of PAD may have been underestimated in this study. Still, this study has some strengths—the overall sample size was not small, the study population was well-defined, a standardised questionnaire and well-recognised assessment tools were used (Edinburgh Questionnaire, ABI), and the study findings were consistent with the published literature. It also provides hard-to-find data on Asian patients. Overall, our study techniques were robust and provided important information for PAD among Asian patients with IS, CVD or DM.

## 5. Conclusions

This study detected PAD by ABI in a quarter of high-risk patients with IS, CVD, or DM. Consistent with the published literature, PAD was associated with increasing age, diabetes mellitus and hypercholesterolemia. Different rates were found within sub-groups and IS, CVD and DM. More such studies on PAD among Asian patients are needed to help fashion PAD management guidelines developed for the Asia–Pacific region [40]. On a practical basis, screening for PAD by ABI should be routinely performed among patients with ischaemic stroke, cardiovascular disease or diabetes mellitus. 

## Figures and Tables

**Table 1:** Characteristics of study subjects overall and by index disease.

Characteristic	Overall (n = 450)	Ischaemic Stroke (n = 150)	Cardiovascular Disease (n = 150)	Diabetes Mellitus (n = 150)	*p*-Value
Age, years mean ± SD	61.9 ± 10.3	62.7 ± 10.2	61.2 ± 11.3	61.7 ± 9.5	0.438
Female %	43.1	44.7	30.7	54.0	<0.001
BMI	23.9 ± 4.3	24.1 ± 4.1	24.0 ± 4.2	23.8 ± 4.5	0.76
Hypertension %	59.3	63.3	56.7	58.0	0.46
Diabetes mellitus %	63.6	43.3	47.3	100.0	<0.001
Number of years of diabetes mellitus if diabetic; years mean ± SD	10.5 ± 9.1	9.8 ± 7.5	10.9 ± 8.4	10.7 ± 10.0	0.76
Hypercholesterolaemia %	39.6	30.0	52.0	36.7	<0.001
Smoker %	42.9	38.0	54.0	36.7	<0.001
Previous stroke %	14.9	18.7	7.3	18.7	0.006
Previous myocardial infarction %	11.3	3.3	18.0	12.7	0.001
Intermittent claudication %	18.4	16.0	15.3	24.0	0.098
PAD by Edinburgh Questionnaire %	11.1	12.0	8.7	12.7	0.26
PAD by ABI %	27.1	22.0	29.3	30.0	0.224

Legend—SD, standard deviation; ABI, ankle brachial index.

**Table 2:** Peripheral disease in index disease subtypes.

Disease	Subtype	PAD Present (n)	PAD Absent (n)	*p*
Ischaemic stroke	Lacunar	16	81	0.03
	Non-lacunar	17	13	
Cardiovascular disease	Unstable angina	15	44	0.56
	ST elevation myocardial infarction	11	28	
	Non-ST elevation myocardial infarction	18	34	
Diabetes mellitus	Insulin-dependant	3	2	0.14
	Non-insulin dependant	42	103	

**Table 3:** Factors associated with peripheral artery disease, univariable, and multivariable analyses.

Characteristic	PAD Present (n = 122)	PAD Absent (n = 328)	*p*-Value	OR	95% CI	*p*-Value
Age, years mean ± SD	65.1 ± 10.1	60.65 ± 10.2	0.001	1.04/year	1.01–1.06	<0.001
Female %	52.5	39.6	0.015	1.50	0.96–2.33	0.07
BMI	23.1 ± 4.5	24.3 ± 4.1	0.014	0.94	0.89–0.99	0.026
Hypertension %	61.5	58.5	0.57			
Diabetes mellitus %	75.4	59.1	0.001	1.95	1.20–3.18	0.007
Number of years of diabetes mellitus if diabetic; years mean ± SD	11.8 ± 9.2	9.9 ± 8.9	0.094			
Hypercholesterolemia %	50.0	35.7	0.006	1.82	1.17–2.82	0.007
Smoker %	41.0	43.6	0.62			
Index disease			0.224			
Ischaemic stroke	27.0	35.7				
Cardiovascular disease	36.1	32.3				
Diabetes mellitus	36.9	32.0				
Intermittent claudication %	30.3	14.0	<0.001			
PAD by Edinburgh Questionnaire %	19.7	7.9	<0.001			

Legend—PAD, peripheral vascular disease; OR, odds ratio; SD, standard deviation.

## Data Availability

Data are available from Tan Tock Seng Hospital.

## References

[1] Hennion D.R., Siano K.A. (2013). Diagnosis and treatment of peripheral arterial disease. Am. Fam. Physician.

[2] Eid M.A., Mehta K., Barnes J.A., Wanken Z., Columbo J.A., Stone D.H., Goodney P., Smith M.M. (2023). The global burden of peripheral artery disease. J. Vasc. Surg..

[3] Song P., Rudan D., Zhu Y., Fowkes F.J.I., Rahimi K., Fowkes F.G.R., Rudan I. (2019). Global, regional, and national prevalence and risk factors for peripheral artery disease in 2015: An updated systematic review and analysis. Lancet Glob. Health.

[4] Olinic D.M., Spinu M., Olinic M., Homorodean C., Tataru D.A., Liew A., Schernthaner G.H., Stanek A., Fowkes G., Catalano M. (2018). Epidemiology of peripheral artery disease in Europe: VAS Educational Paper. Int. Angiol..

[5] Selvin E., Erlinger T.P. (2004). Prevalence of and risk factors for peripheral arterial disease in the United States: Results from the National Health and Nutrition Examination Survey, 1999–2000. Circulation.

[6] You Y., Wang Z., Yin Z., Bao Q., Lei S., Yu J., Xie X. (2023). Global disease burden and its attributable risk factors of peripheral arterial disease. Sci. Rep..

[7] Ouriel K. (2001). Peripheral arterial disease. Lancet.

[8] Abramson B.L., Al-Omran M., Anand S.S., Albalawi Z., Coutinho T., de Mestral C., Dubois L., Gill H.L., Greco E., Guzman R. (2022). Canadian Cardiovascular Society 2022 Guidelines for Peripheral Arterial Disease. Can. J. Cardiol..

[9] Aboyans V., Ricco J.B., Bartelink M.E.L., Björck M., Brodmann M., Cohnert T., Collet J.P., Czerny M., De Carlo M., Debus S. (2018). 2017 ESC Guidelines on the Diagnosis and Treatment of Peripheral Arterial Diseases, in collaboration with the European Society for Vascular Surgery (ESVS): Document covering atherosclerotic disease of extracranial carotid and vertebral, mesenteric, renal, upper and lower extremity arteries. Endorsed by: The European Stroke Organization (ESO)The Task Force for the Diagnosis and Treatment of Peripheral Arterial Diseases of the European Society of Cardiology (ESC) and of the European Society for Vascular Surgery (ESVS). Eur. Heart J..

[10] Gerhard-Herman M.D., Gornik H.L., Barrett C., Barshes N.R., Corriere M.A., Drachman D.E., Fleisher L.A., Fowkes F.G.R., Hamburg N.M., Kinlay S. (2017). 2016 AHA/ACC Guideline on the Management of Patients with Lower Extremity Peripheral Artery Disease: A Report of the American College of Cardiology/American Heart Association Task Force on Clinical Practice Guidelines. J. Am. Coll. Cardiol..

[11] Bauersachs R., Debus S., Nehler M., Huelsebeck M., Balradj J., Bowrin K., Briere J.-B. (2020). A Targeted Literature Review of the Disease Burden in Patients With Symptomatic Peripheral Artery Disease. Angiology.

[12] Gutierrez J.A., Mulder H., Jones W.S., Rockhold F.W., Baumgartner I., Berger J.S., Blomster J.I., Fowkes F.G.R., Held P., Katona B.G. (2018). Polyvascular Disease and Risk of Major Adverse Cardiovascular Events in Peripheral Artery Disease: A Secondary Analysis of the EUCLID Trial. JAMA Netw. Open.

[13] Alberts M.J., Bhatt D.L., Mas J.-L., Ohman E.M., Hirsch A.T., Röther J., Salette G., Goto S., Smith S.C., Liau C.-S. (2009). REduction of Atherothrombosis for Continued Health Registry Investigators. Three-year follow-up and event rates in the international REduction of Atherothrombosis for Continued Health Registry. Eur. Heart J..

[14] Kullo I.J., Rooke T.W. (2016). Peripheral Artery Disease. N. Engl. J. Med..

[15] Leng G.C., Fowkes F.G. (1992). The Edinburgh Claudication Questionnaire: An improved version of the WHO/Rose Questionnaire for use in epidemiological surveys. J. Clin. Epidemiol..

[16] Aboyans V., Criqui M.H., Abraham P., Allison M.A., Creager M.A., Diehm C., Fowkes F.G., Hiatt W.R., Jönsson B., Lacroix P. (2012). Measurement and interpretation of the ankle-brachial index: A scientific statement from the American Heart Association. Circulation.

[17] Herraiz-Adillo Á., Cavero-Redondo I., Álvarez-Bueno C., Pozuelo-Carrascosa D.P., Solera-Martínez M. (2020). The accuracy of toe brachial index and ankle brachial index in the diagnosis of lower limb peripheral arterial disease: A systematic review and meta-analysis. Atherosclerosis.

[18] Bennett P.C., Silverman S., Gill P.S., Lip G.Y. (2009). Ethnicity and peripheral artery disease. QJM.

[19] Hobbs S.D., Wilmink A.B., Bradbury A.W. (2003). Ethnicity and peripheral arterial disease. Eur. J. Vasc. Endovasc. Surg..

[20] Vitalis A., Lip G.Y., Kay M., Vohra R.K., Shantsila A. (2017). Ethnic differences in the prevalence of peripheral arterial disease: A systematic review and meta-analysis. Expert Rev. Cardiovasc. Ther..

[21] Sebastianski M., Makowsky M.J., Dorgan M., Tsuyuki R.T. (2014). Paradoxically lower prevalence of peripheral arterial disease in South Asians: A systematic review and meta-analysis. Heart.

[22] Amudha K., Chee K.H., Tan K.S., Tan C.T., Lang C.C. (2003). Prevalence of peripheral artery disease in urban high-risk Malaysian patients. Int. J. Clin. Pract..

[23] Golledge J., Moxon J.V., Rowbotham S., Pinchbeck J., Quigley F., Jenkins J. (2020). High ankle brachial index predicts high risk of cardiovascular events among people with peripheral artery disease. PLoS ONE.

[24] Yang Y., Liu L., Sun H., Nie F., Hu X. (2020). Relation between high Ankle-Brachial Index and cardiovascular outcomes in the general population and cardiovascular disease: A meta-analysis. Int. Angiol..

[25] Singhania P., Das T.C., Bose C., Mondal A., Bhattacharjee R., Singh A., Mukhopadhyay S., Chowdhury S. (2024). Toe brachial index and not ankle brachial index is appropriate in initial evaluation of peripheral arterial disease in type 2 diabetes. Diabetol. Metab. Syndr..

[26] Chung P.-W., Kim D.-H., Kim H.Y., Park K.-Y., Park T.H., Hong J.M., Kim G.-M., Bang O.Y., Oh K., Lee S.J. (2013). Differences of ankle-brachial index according to ischemic stroke subtypes: The peripheral artery disease in Korean patients with ischemic stroke (PIPE) study. Eur. Neurol..

[27] Rahman A.S., Akhtar S.W., Jamal Q., Sultana N., Siddiqui M.A., Hassan Z. (2017). Ischaemic stroke and peripheral artery disease. J. Pak. Med. Assoc..

[28] Ratanakorn D., Keandoungchun J., Tegeler C.H. (2012). Prevalence and association between risk factors, stroke subtypes, and abnormal ankle brachial index in acute ischemic stroke. J. Stroke Cerebrovasc. Dis..

[29] Kim E.K., Song P.S., Yang J.H., Bin Song Y., Hahn J.-Y., Choi J.-H., Gwon H.-C., Lee S.H., Hong K.P., Park J.E. (2013). Peripheral artery disease in korean patients undergoing percutaneous coronary intervention: Prevalence and association with coronary artery disease severity. J. Korean Med. Sci..

[30] Dharmasaroja P.A., Piyayotai D., Hutayanon P., Buakhamsri A., Intharakham K. (2010). Extracranial carotid stenosis and peripheral arterial disease in Thai patients with coronary artery disease. Angiology.

[31] Sim E.K., Koo G., Adebo O.A., Lim M.C., Choo M.H., Lee C.N. (1993). Prevalence of peripheral artery disease in patients with coronary artery disease. Ann. Acad. Med. Singap..

[32] Rhee S.Y., Guan H., Liu Z.M., Cheng S.W.-K., Waspadji S., Palmes P., Tai T.Y., Suwanwalaikorn S., Kim Y.S. (2007). PAD-SEARCH Study Group. Multi-country study on the prevalence and clinical features of peripheral arterial disease in Asian type 2 diabetes patients at high risk of atherosclerosis. Diabetes Res. Clin. Pract..

[33] Maeda Y., Inoguchi T., Tsubouchi H., Sawada F., Sasaki S., Fujii M., Saito R., Yanase T., Shimabukuro M., Nawata H. (2008). High prevalence of peripheral arterial disease diagnosed by low ankle-brachial index in Japanese patients with diabetes: The Kyushu Prevention Study for Atherosclerosis. Diabetes Res. Clin. Pract..

[34] Lekshmi Narayanan R.M., Koh W.P., Phang J., Subramaniam T. (2010). Peripheral arterial disease in community-based patients with diabetes in Singapore: Results from a Primary Healthcare Study. Ann. Acad. Med. Singap..

[35] Li Z., Liu J. (2014). Coexistence of low ankle-brachial index and intra-cranial atherosclerosis?. Int. Angiol..

[36] Mourad J.-J., Cacoub P., Collet J.-P., Becker F., Pinel J.-F., Huet D., Sevestre-Pietri M.-A., Priollet P. (2009). ELLIPSE scientific committee and study investigators. Screening of unrecognized peripheral arterial disease (PAD) using ankle-brachial index in high cardiovascular risk patients free from symptomatic PAD. J. Vasc. Surg..

[37] Lempesis I.G., Varrias D., Sagris M., Attaran R.R., Altin E.S., Bakoyiannis C., Palaiodimos L., Dalamaga M., Kokkinidis D.G. (2023). Obesity and Peripheral Artery Disease: Current Evidence and Controversies. Curr. Obes. Rep..

[38] Huang Y., Xu M., Xie L., Wang T., Huang X., Lv X., Chen Y., Ding L., Lin L., Wang W. (2016). Obesity and peripheral arterial disease: A Mendelian Randomization analysis. Atherosclerosis.

[39] Sritara P., Sritara C., Woodward M., Wangsuphachart S., Barzi F., Hengprasith B., Yipintsoi T. (2007). Prevalence and risk factors of peripheral arterial disease in a selected Thai population. Angiology.

[40] Abola M.T.B., Golledge J., Miyata T., Rha S.-W., Yan B.P., Dy T.C., Ganzon M.S.V., Handa P.K., Harris S., Zhisheng J. (2020). Asia-Pacific Consensus Statement on the Management of Peripheral Artery Disease: A Report from the Asian Pacific Society of Atherosclerosis and Vascular Disease Asia-Pacific Peripheral Artery Disease Consensus Statement Project Committee. J. Atheroscler. Thromb..

